# Subsentence Extraction from Text Using Coverage-Based Deep Learning Language Models

**DOI:** 10.3390/s21082712

**Published:** 2021-04-12

**Authors:** JongYoon Lim, Inkyu Sa, Ho Seok Ahn, Norina Gasteiger, Sanghyub John Lee, Bruce MacDonald

**Affiliations:** 1 CARES, Department of Electrical, Computer and Software Engineering, University of Auckland, Auckland 1142, New Zealand; jy.lim@auckland.ac.nz (J.L.); hs.ahn@auckland.ac.nz (H.S.A.); ngas906@aucklanduni.ac.nz (N.G.); sanghyub.lee@auckland.ac.nz (S.J.L.); b.macdonald@auckland.ac.nz (B.M.); 2 CSIRO Data61, Robotics and Autonomous Systems Group, Perception Group, Pullenvale 4069, Australia

**Keywords:** sentiment analysis, text extraction, span prediction, natural language processing, human robot interaction, bidirectional transformer

## Abstract

Sentiment prediction remains a challenging and unresolved task in various research fields, including psychology, neuroscience, and computer science. This stems from its high degree of subjectivity and limited input sources that can effectively capture the actual sentiment. This can be even more challenging with only text-based input. Meanwhile, the rise of deep learning and an unprecedented large volume of data have paved the way for artificial intelligence to perform impressively accurate predictions or even human-level reasoning. Drawing inspiration from this, we propose a coverage-based sentiment and subsentence extraction system that estimates a span of input text and recursively feeds this information back to the networks. The predicted subsentence consists of auxiliary information expressing a sentiment. This is an important building block for enabling vivid and epic sentiment delivery (within the scope of this paper) and for other natural language processing tasks such as text summarisation and Q&A. Our approach outperforms the state-of-the-art approaches by a large margin in subsentence prediction (i.e., Average Jaccard scores from 0.72 to 0.89). For the evaluation, we designed rigorous experiments consisting of 24 ablation studies. Finally, our learned lessons are returned to the community by sharing software packages and a public dataset that can reproduce the results presented in this paper.

## 1. Introduction

Understanding human emotion or sentiment is one of the most complex and active research areas in physiology, neuroscience, neurobiology, and computer science. Liu and Zhang [1] defined sentiment analysis as the computational study of people’s opinions, appraisals, attitudes, and emotions toward entities, individuals, issues, events, topics and their attributes. The importance of sentiment analysis is gaining momentum in both individual and commercial sectors. Customers’ patterns and emotional states can be derived from many sources of information (e.g., reviews or product ratings), and precise analysis of them often leads to direct business growth. Sentiment retrieval from personal notes or extracting emotions in multimedia dialogues generates a better understanding of human behaviours (e.g., in crime or abusive chat). Sentiment analysis and emotion understanding have been applied to the field of Human–Robot Interaction (HRI) in order to develop robotics that can form longer-term relationships and rapport with human users by responding with empathy. This is crucial for maintaining interest in engagement when the novelty wears off. Additionally, robots that can identify human sentiment are more personalized, as they can adapt their behaviour or speech according to the sentiment. The importance of personalization in HRI is widely reported and impacts cooperation [2], overall acceptability [3,4] and interaction and engagement [2,5]. Indeed, an early review by Fong [6] on characteristics of successful socially interactive robots suggested that emotions, dialogue, and personality are crucial to facilitating believable HRI.

Sentiment can be efficiently detected by exploiting multimodal sensory information such as voice, gestures, images, or heartbeats, as evident in well-established literature and studies in physiology and neurosciences [7]. However, reasoning sentiment from text information such as tweets, blog posts, and product reviews is a more difficult problem. This is because text often contains limited information to describe emotions sufficiently. It also differs across gender, age, and cultural backgrounds [8]. Furthermore, sarcasm, emojis, and rapidly emerging new words restrict sentiment analysis from text data. Conventional rule-based approaches may fail in coping with these dynamic changes.

**Figure 1:**
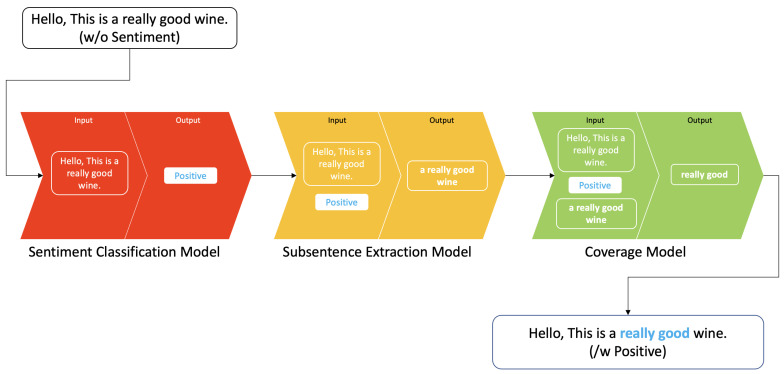
Processing pipeline with an example input sentence. There are three cascaded models to predict both sentiment and subsentence. Each model consists of a bidirectional transformer model with varying output layers.

To address these challenges, we present a study that adopts the latest technologies developed in artificial intelligence (AI) and natural language processing (NLP) for sentiment and subsentence extraction, as shown in Figure 1. The recent rise of AI with deep neural networks and massively big data have demonstrated super human-level performance in many tasks including image classification and object detection, cyber-security, entertainment (e.g., playing GO or DOTA), and NLP (e.g., Q&A [9]). Sentiment detection from text is one of the subsets of NLP. It is convincing that these language models can adequately capture the underlying sentiment from text data (more details are presented in Section 2).

This paper presents a sentiment classification and subsentence extraction model from text input using deep neural networks and shows an improvement of the extraction accuracy with a coverage-based span prediction model. Therefore the contributions of this paper, are

- Proposing a novel coverage-based subsentence extraction system that takes into considerations the length of subsentence and an end-to-end pipeline that can be directly leveraged by Human-Robot Interaction;
- Performing intensive qualitative and quantitative evaluations of sentiment classification and subsentence extraction
- Returning lessons learnt to the community as a form of open dataset and source code (https://github.com/UoA-CARES/BuilT-NLP, accessed on 20 April 2021).

The rest of the paper is structured as follows; Section 2 presents the state-of-the-art (SOTA) NLP studies in sentiment analysis and its relevant usage within HRI. Section 3 addresses the detailed approach we proposed, such as coverage-based subsentence extraction and Exploratory Data Analysis (EDA) of the dataset. Section 4 delineates the dataset used in this paper and its preparation for model training that is addressed in Section 5. This is followed by experiments results in sentiment classification and subsentence extraction in Section 6. We discuss the advantages and limitations of the proposed approach in Section 7 to highlight opportunities for future work in this field and conclude the paper by presenting a summary in Section 8.

## 2. Related Work

Sentiment detection is an interdisciplinary task that requires tight connections between multiple disciplines. In this section, we introduce related studies in NLP, sentiment analysis and emotion detection, and HRI.

### 2.1. Natural Language Processing for Sentiment Analysis

There has been a large amount of public and commercial interests in NLP for many decades. The primary objective is to aid machines in understanding human languages by utilising linguistics and computational techniques [10]. In particular, its importance and impact are gaining momentum with the rise of big data and deep learning techniques in interpreting human-level contextual information or generating vivid artificial articles (Generative Pre-trained Transformer 3, GPT-3) [9]. Nowadays, the prominent applications of NLP are language translation [11], text summarisation [12], Q&A tasks [13,14,15,16,17,18], information retrieval [19], and sentiment analysis [20,21,22].

Most of these data-driven approaches require a high fidelity and quality training dataset. There are several publicly available datasets as reported by [7]. These datasets are valuable and cover broad spectrums such as cross-cultural studies, news, tales, narratives, dialogues, and utterances. However, all these datasets only provide an emotion label for each sentence rather than fine-grain subsentence labels that we leverage in this paper. For instance, these datasets only provide positive sentiment for a sentence “Hello this is a really good wine”, whereas we aim to predict both polarity of the sentence and the parts of a sentence that lead to the positive sentiment, i.e., “really good”. In this context, to the author’s best knowledge, the dataset we used is unique and one of the pioneering studies in processing fine-grain sentiment detection.

### 2.2. Emotion Detection in Sentiment Analysis

Emotion detection is a subset of sentiment analysis that seeks not only polarity (e.g., positive, negative, or neutral) from input sentence or speech but tries to derive more detailed emotions (e.g., happy, sad, anxious, nervous instead positive or negative). Regarding this topic, there is an interesting survey report [7]. This comprehensive survey focuses on conventional rule-based and current deep learning-based approaches. There are two key points from this article: it highlights the use of the bidirectional transformer model [23] that boosts overall classification performance by a large margin, and it lacks adequate application of emotion detection. These points are well aligned with our approach and goal; we used a variant Bidirectional Encoder Representations from Transformers (BERT) language model [24] as a backbone model and proposed a practical application in HRI. This may reflect that the approach and the research goal of this paper are well-defined. We believe that the proposed method can be exploited in many relevant domains such as daily human–machine interaction (e.g., Alexa or Google Assistant), mental care services (e.g., depression treatment), or in aged care.

Sentiment analysis is a sophisticated interdisciplinary field that connects linguistic and text mining with AI, predominantly for NLP [25]. Dominant sentiment analysis strategies may include emotion detection, subjectivity detection, or polarity classification (e.g., classifying positive, negative and neutral sentiments [26]). These techniques require an understanding of complicated human psychology. For instance, they may include instant and short-lasting emotions [27], longer-lasting and unintentional moods [28], and feelings or responses to emotions [25].

### 2.3. Human–Robot Interaction

The importance of personalisation in HRI is widely reported, and some experiments have been conducted to compare robotic systems with and without sentiment abilities, including expressing and analysing sentiment [25,29,30,31,32]. These studies used the NAO, Kismet, and Reeti robots. An early and well-known study by Breazeal [29] recruited five participants to interact with Kismet in different languages. The individuals understood the robot’s emotions successfully. Additionally, humans and the robot mirrored one another, demonstrating a phenomenon called affective mirroring. This psychological phenomenon is often observed in human-to-human interaction, whereby an emotion is subconsciously mirrored. This was evidenced in the experiment, whereby participants apologised and looked to the researcher with anguish, displaying guilt or stating that they felt terrible when they made Kismet feel sad.

In three experimental studies, the robots were evaluated when completing the task of reading fables/stories. The purpose of these experiments was to determine the effect of emotion in HRI. In one example, Striepe [30] recruited 63 German participants aged 18 to 30 years and assigned them to one of three groups: audiobook story, emotional social robot storyteller, or neutral robot storyteller. They concluded that the emotional robot storyteller was just as effective at “transporting” listeners into the story as the audiobook. The neutral robot performed the worst. Similar findings were found by Szabóová [25], who reported that their robot with emotion expression, for example, voice pitch, was better rated overall. Participants even thought that the robot appeared capable of understanding, compared to the neutral robot. These findings also transferred to other activities, such as gaming. Shen [31] developed a robot that mimicked user’s facial expressions and one that demonstrated sentiment apprehension, for example, a robot’s ability to reason about the user’s attitudes such as judgment/liking. They found that people wanted to play games on the robot with sentiment apprehension more than the other, as well as rated it to be more engaging. Evidently, sentiment plays an important role in HRI, including in simulating human phenomena and perceptions, for example, understanding and affective mirroring and being superior to their neutral counterparts.

Some observational (non-experimental) research has also explored the effectiveness of emotion expression by robots. For example, in Rodriguez [33], 26 nine-year-old children interacted with a robot to determine whether they could understand the robot’s emotional expression. The authors concluded that the children could understand when the robot was expressing sad or happy emotions by its facial expressions and gestures (e.g., fast and slow movements or changes of lights to its eyes). Additionally, in a small pilot study with four participants, an emotionally expressive storytelling robot attempted to persuade listeners to make a decision, using facial expressions and head movements [34]. The authors conclude that an amended version of this robot may potentially increase the motivation, interest and engagement of users in the task.

From the literature review, we have shown that deep learning transformer language models’ utilisation is one of the main streams in text-based sentiment extraction. Besides, there exists a massive demand for such systems in various applications mentioned above (e.g., HRI). However, there are still remaining challenges, such as the lack of a unified sentiment extraction pipeline that can be directly applied to existing systems and the need for a high-performance sentiment extraction model.

We propose coverage-based subsentence extraction and an easy-to-use processing pipeline in the following sections to address these challenges.

## 3. Methodologies

In this section, we present the methodologies used later in this paper, including Bidirectional Encoder Representations from Transformers (BERT) and a Robustly Optimized BERT Pretraining Approach (RoBERTa) NLP models followed by sentiment classification and coverage-based subsentence extraction.

### 3.1. BERT and RoBERTa Transformer Language Models

Our proposed model is built on RoBERTa [24], which is one of the variants of BERT [23] that improves overall performance (e.g., F1 scores) in language understanding and Q&A tasks by taking into account dynamic masking, full-sentences without Next Sentence Prediction (NSP) loss, and a larger byte-level Byte-Pair Encoding (BPE) with additional hyper-parameter tuning (e.g., batch size). With the superior performance of RoBERTa over BERT, another selection criterion is that it makes use of dynamic masking, which can efficiently handle high-variance daily-life text such as tweets, text messages, or daily dialogues. Technical details and deeper explanations can be found in the original papers [23,24]. We therefore only provide a summary and high-level overview for contextual purposes.

BERT is considered one of the most successful language models for NLP proposed by the Google AI language team in 2018. As the name implies, the fundamental idea is to take account of both sequential directions (i.e., left-to-right and right-to-left) in order to capture more meaningful contextual information. Inside of these models, an attention-driven language learning model, Transformer [35] (only the encoder part), is exploited in our method to extract context with Next Sentence Prediction (NSP) and Masked Language Model (MLM). Varying pre-trained BERT models exist depending on the number of encoders, such as BERT-base (12 encoders, 110M parameters) or BERT-large (24 encoders, 330M parameters), with the larger model often producing better results but taking more training time.

**Figure 2:**
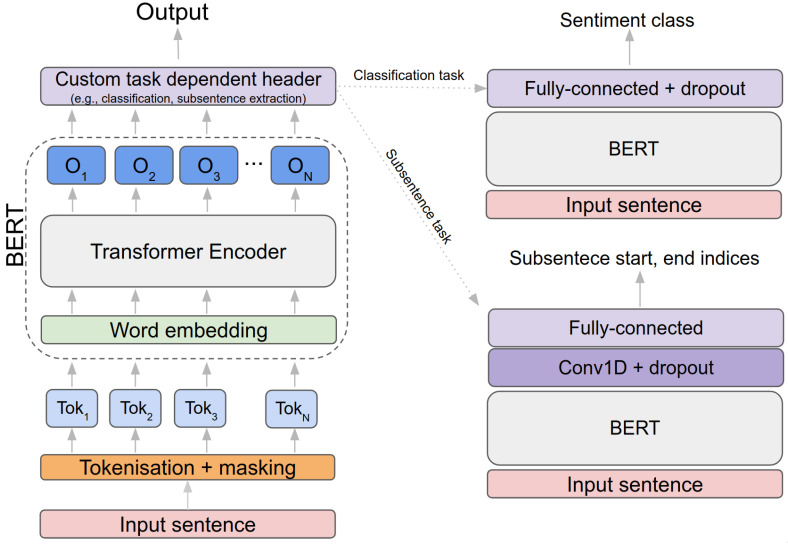
An overview of Bidirectional Encoder Representations from Transformers (BERT) (**left**) and task-driven fine-tuning models (**right**). Input sentence is split into multiple tokens ($Tok_{N}$) and fed to a BERT model, which outputs embedded output feature vectors, $O_{N}$, for each token. By attaching different head layers on top, it transforms BERT into a task-oriented model.

NSP plays a role in distinguishing whether two input sentences are highly correlated or not by formulating a binary classification problem (e.g., if two adjacent sentences are sampled from the same dataset, this pair is treated as positive samples, and otherwise as negative samples). MLM is performed by randomly masking words from the input sentence with [MASK] (e.g., “Today is a great day” can be “Today is a [MASK] day”), and the objective of MLM is the correct prediction of these masked words. Figure 2 illustrates the overall pipeline and BERT’s pre-training and fine-tuning procedures. For more technical detail, please refer to the original paper [23].

**Figure 3:**
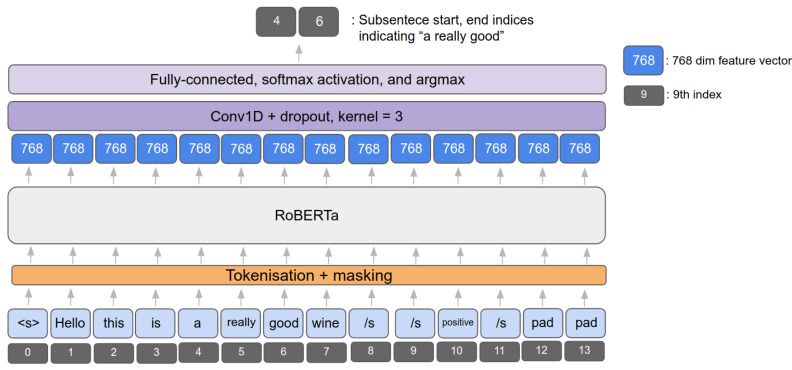
This diagram exemplifies how input sentence is processed using Robustly Optimized BERT Pretraining Approach (RoBERTa) model. Gray indicates a token index and blue is embedded output feature vector.

RoBERTa proposes a couple of network design choices and BERT training strategies such as adopting dynamic masking and removing NSP, which may degrade the performance in specific downstream tasks. In addition to these, intensive hyper-parameter tuning and ablation studies have been conducted with various tasks and public datasets. Dynamic masking is more suitable for our task of dealing with high-variance datasets such as tweets or daily conversations. Drawing inspiration from this, we decided to build our baseline model upon RoBERTa. Figure 3 illustrates sentiment inference task using a pre-trained ${\mathrm{BERT}}_{base}$ model. A positive contextual sentence (i.e., tokenised, covered this in the next section) is fed into the model, creating 768 feature vectors for each token (by feature embedding). It is then processed throughout the custom headers such as 1D Convolution followed by Softmax activation. The final output then indicates that the subsentence from 4th to 6th token (i.e., “a really good”) is predicted from the network.

### 3.2. Sentiment Classification

Given the transformers model mentioned earlier, we are interested in extracting subsentences from an input sentence and estimating the sentiment of the sentence. This is mainly because this information can significantly boost the subsentence extraction prediction by narrowing down the potential search space. It is also useful for other applications such as tone estimation or emotion detection. This problem is well-formulated as supervised learning, meaning that we provide a known label (e.g., positive, negative, or neutral) for a sentence. A neural network is asked to predict the corresponding label.

Although we only consider three classes in this paper, there are no limitations to the number of classes as long as the class label is provided. We design a simple neural network with two additional layers (i.e., dropout and Fully-connected layer) on top of the RoBERTa model. The overall sentiment classification network architecture is shown in Figure 2, and the relevant results are presented in Section 6.3.

### 3.3. Coverage Model for Subsentence Extraction

Transformer models have been widely used for NLP, image-based classification, and object detection tasks [36]. These attention-based transformer networks can be improved by providing additional metadata. Sentiment information and attention mask, for example, are useful sources for subsentence extraction. Furthermore, extracting efficient features such as a span of subsentence or character-level encoding instead of word-level encoding can also improve the model’s performance.

Within this context and drawing inspiration from [37], we propose a recursive approach that estimates the length of subsentence in an input sentence. We refer tp the length of subsentence as coverage, *c*, which is a scalar and computed as $c=\frac{M}{N}\times \kappa$, where *M* and *N* are the length of subsentence and input sentence, respectively. κ is a scale parameter governing the width of *c*. We empirically initialised this as 15. It is worth mentioning that the overall performance is more or less independent of this, if and only if *c* is larger than a threshold. This is because *c* will decrease regardless of an initial value. However, we found that if *c* was too small, then the model struggled to find the correct subsentence because our model could not expand the coverage range. Regarding this issue, we discuss more detail in the future work and limitations (Section 7).

**Figure 4:**
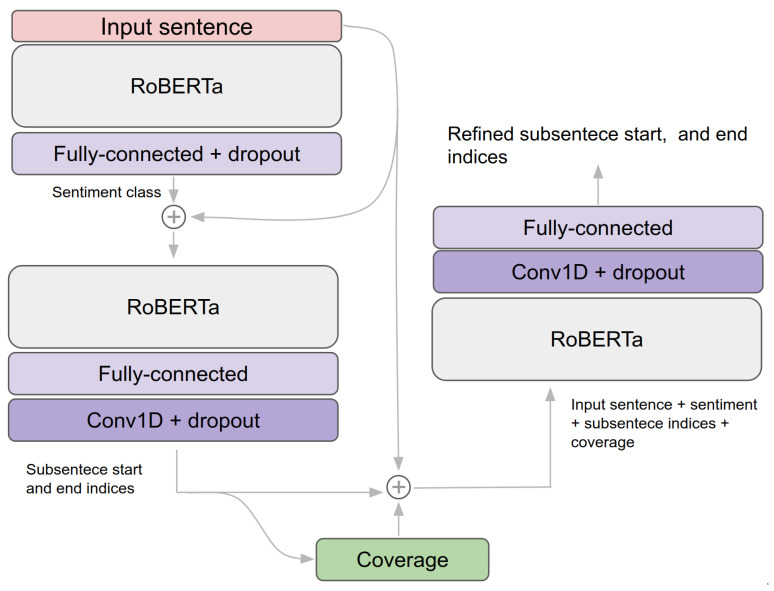
Coverage-base subsentence extraction network architecture and pipeline. This is an in-depth representation of Figure 1 with data flow.

In summary, our coverage model takes the following steps: (1) predicting the length of subsentence (i.e., coverage) by utilising a transformer network that outputs start and end indices (e.g., Q&A or text summarisation networks); (2) computing coverage and feeding back into a coverage model with an input sentence and previously predicted indices. For more detail and better understanding, Algorithm 1 delineates model pseudo code and high-level data flow is shown in Figure 4.
**Algorithm 1:** Coverage-based subsentence extraction algorithm.
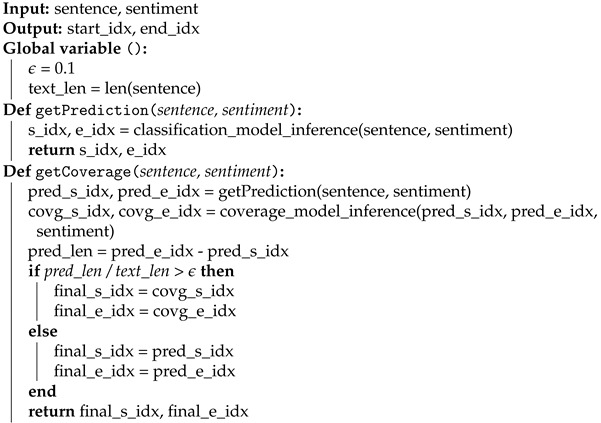


### 3.4. End-To-End Sentiment and Subsentence Extraction Pipeline

Given classifier and coverage models from the previous sections, we propose an end-to-end pipeline capable of simultaneously extracting sentiment and subsentence from an input text. The pipeline architecture is straightforward and is formed by a series of models implying that each model’s outputs are fed to the following subsequent model’s inputs. We present more detail and discussion on the experimental results in Section 6.5.

## 4. Dataset and Experiments Design

In this section, we provide explanations of the dataset we used and the design of our experiments. Furthermore, input data preprocessing and tokenisation details are presented with Exploratory Data Analysis.

**Figure 5:**
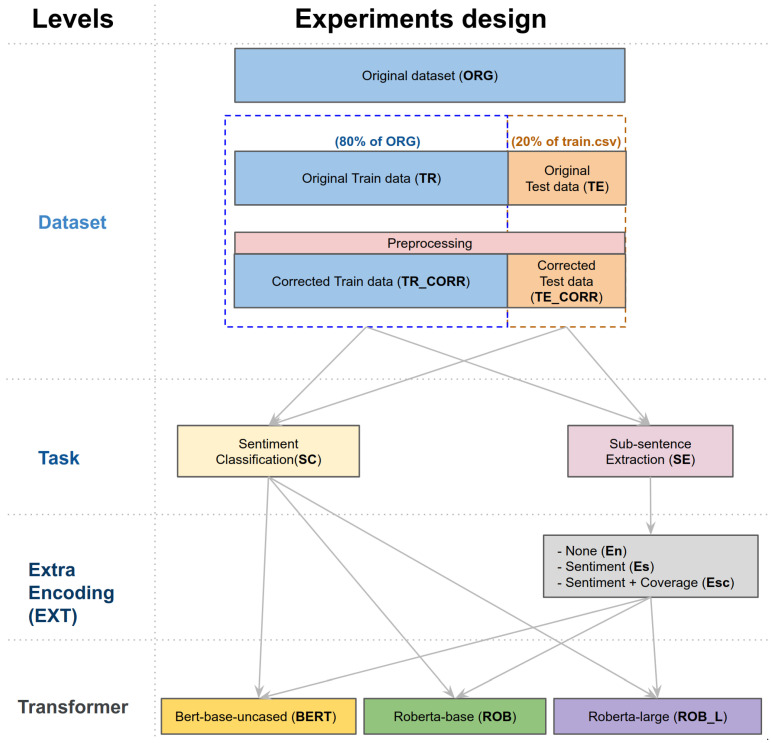
Experiment design. This diagram shows the experiment design scheme proposed in this manuscript. There are total 24 experiments named by the following convention: [Dataset]_[Task]_[EXT]_[Transformer]. For example, 21.[TR_CORR]_[SE]_[Esc]_[ROB] indicates the experiment for Subsentence extraction task with sentiment + coverage options trained on the corrected train data with RoBERTa-base model. The prefixed number is a unique experiment ID for convenience.

As shown in Figure 5, we define four levels: dataset, task, extra encoding, and transformer. Dataset level refers to how we split and organise, train, validate, and test the dataset. At high-level perspectives, we use 80% of the original dataset to train and 20% for the test. Note that we utilised five folds stratified Cross-Validation (CV) of the train set, meaning that the validation set takes about 16% (i.e., one fold) and the actual training set is about 64%. More detail regarding this is in Section 5.2. We define acronyms to stand for training and testing the dataset as **TR** and **TE**. The postfix **CORR** indicates the corrected dataset by preprocessing.

Task level has two categories: sentiment classification (**SC**) and subsentence extraction (**SE**). While the classification is directly connected to a transformer model, **SE** has three extra encoding variations: None (**En**), Sentiment (**Es**), and Sentiment with coverage (**Esc**). **En** indicates subsentence extraction without any extra encoding data, **Es** and **Esc** perform the task with sentiment and sentiment + coverage metadata, respectively.

Finally, the transformer level has three variations that we introduced in the earlier section; **BERT**, **ROB** (12 encoders, 110M parameters), and **ROB_L** (24 encoders, 330M parameters).

These four levels create 24 unique combinations as shown in Table 1 with the naming conventions such that [Dataset]_[Task]_[EXT]_[Transformer]. For instance, 15.[TR]_[SE]_[Esc]_[ROB_L] implies the experiment for subsentence extraction task with sentiment + coverage options trained on the original training dataset with the RoBERTa-large model. These notations will be consistently used across the paper.

### 4.1. Tokenisation and Input Data Preprocessing

This section describes the input data preprocessing idea and tokenisation that we performed before model training.

The preprocessing objective is to uniformly format and clean input data so that the subsequent tokeniser and model will be able to interpret efficiently. Therefore, preprocessing is one of the important procedures of NLP for training well-generalised and high-performance language models. There are many preprocessing techniques such as lower-casing, removing unnecessary characters (extra white-spaces, special characters, HTML tags), or lemmatisation (stemming of words, e.g., cars or car’s will be converted to car). To address all possible cases is a non-trivial task due to high complexity and large diversity in natural language; thus, we simply yet effectively applied lowercase to all texts and removed URL and HTML tags from the original input training data. In addition, the original dataset contains meaningless full stops (e.g., “...” or “..”), which are trivial in sentimental perspectives but significantly affect sentiment classification results and subsentence extraction performance. Therefore, we replaced them with a single stop. We will discuss more of this topic in the experimental section.

Followed by special character cleaning, we also performed tokenisation, converts words into either characters or subwords, as it is considered one of the most important steps in NLP. In essence, the idea is to split the word into meaningful pieces and map between an input word and the corresponding digits (so-called encoding). This is shown in Table 2. For example, from the Figure, we have an input sentence, “Hello this is a really good wine” with the subsentence that is a manual label justifying why this sentence is labelled as a positive sentiment. Then, **tokens** and **input_ids** show tokenised words and the corresponding encoding. Note that there is a specific input format of RoBERTa, which is originally designed for a question and answer task such as starting with <s> tag followed by question tokens and adding stop tags with answer tokens at the end. For the **attention_mask** we set all as true because we want the model to focus on all tokens except padding. **start_token** and **end_token** indicate the start and end index of the selected text. RoBERTa makes use of GPT-2 tokeniser, using byte-level BPE [38], and we utilise the popular pre-trained RoBERTa tokeniser from huggingface (https://huggingface.co/transformers/model_doc/roberta.html, accessed on 11 April 2021).

### 4.2. Tweet Sentiment Dataset

We used a public dataset from the Kaggle tweet sentiment extraction competition (https://www.kaggle.com/c/tweet-sentiment-extraction/data, accessed on 11 April 2021). Among many other publicly available datasets such as ISEAR (https://www.kaggle.com/shrivastava/isears-dataset, accessed on 11 April 2021) or SemEval-2017 (https://alt.qcri.org/semeval2017/task4/, accessed on 11 April 2021), this dataset is unique because it contains not only the sentiment of sentences (e.g., positive, negative, or neutral) but more importantly “selected text” which are words or phrases drawn from original tweets. The total number of samples in this dataset is about 27k, and detail is provided in Table 3. This “selected text” is why the corresponding sentence holds its sentiment, as shown in Table 4.

Table 4 shows the top 5 row-wise samples from the dataset (i.e., each row indicates one instance). Each row has five columns; “textID” is a unique ID for each piece of text, “text” is the text of the tweet, “selected text” is the text that supports the tweet’s sentiment, and “sentiment” is the general sentiment of the tweet. We further discuss the dataset in terms of Exploratory Data Analysis (EDA) and data preparation for model training in the following sections.

### 4.3. Exploratory Data Analysis (EDA)

Nowadays, there is well-established knowledge and resources in machine learning or data-driven approaches such as highly-engineered frameworks; Tensorflow, PyTorch, or advanced-tools; SciPy (https://www.scipy.org/, accessed on 11 April 2021); or scikit-learn (https://scikit-learn.org/, accessed on 11 April 2021). Their high-fidelity and powerful feature extraction capability may lead to outstanding performance without any data analysis (just feeding all training data and waiting). However, a high-level understanding of the dataset often plays a crucial role in improving model performance and spotting missing and erroneous data.

In handling sequential information (i.e., in our sentence processing case), correlating words’ contextual meaning and their distribution in training and test sets must be identified prior to model training. Hence, in this section, we provide our EDA strategies and share some underlying insights of the dataset used.

We exploited a public dataset from a tweet-sentiment-extraction competition (https://www.kaggle.com/c/tweet-sentiment-extraction, accessed on 11 April 2021). The organiser provided 27k training samples composed of raw tweet sentence, selected text, and sentiment, as shown in Table 4.

The first column is a unique textID, but this does not provide any useful information. The second and third columns are original tweets and manually labelled texts that determine the type of sentiment in the last column. For example, the second sample, “Sooo SAD I will miss you here in San Diego!!!” is labelled as negative sentiment because of “Sooo SAD” texts. Note that these selected texts (or ground truth) are manually labelled, which can often be subjective, and the organiser randomly sampled training and test sets with some extra noise (e.g., random white spaces in labelling). Although these may result in performance degrading, the dataset is unique and has a sufficiently large volume of samples, so we decided to use this data for model training.

**Figure 6:**
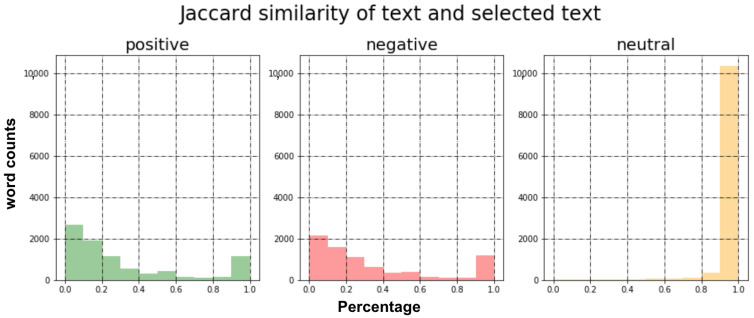
These plots display Jaccard similarity between input sentence and selected subsentence for each sentiment in the dataset. x-axis is the similarity percentage and y-axis is the number of sentences holding the corresponding similarity. Noticeably, sentences with neutral sentiment have the identical subsentence whereas sentences with positive and negative show similar distributions.

It is important to note that there are noises or artifacts in the training dataset. For example, the top row from Table 5 has “onna” as the selected_text with negative sentiment. This sounds irrelevant, and further investigation found that this happens due to *N* exceeding and *M* leading spaces surrounding the selected_text. The original text of the top row is “is␣back␣home␣now␣␣␣␣␣␣gonna␣miss␣every␣one”; we can now see the six invisible leading spaces prior to “gonna”, and our corrected selected text should be “miss” after removing these six leading spaces. We explicitly calculated *N* and *M* and found that there were 1112 cases where selected_text mismatching happened in positive and negative samples (i.e., 14.7%). Note that we did not correct neutral samples since their text and selected_text are almost identical, as shown in Figure 6.

This corrected dataset will be exploited as the baseline dataset across this manuscript, and we will revisit this later in Section 3.3. The similarity metric used in this paper (i.e., Jaccard score and Area-Under-the Curve, AUC) will be presented in the following experiment results section.

**Figure 7:**
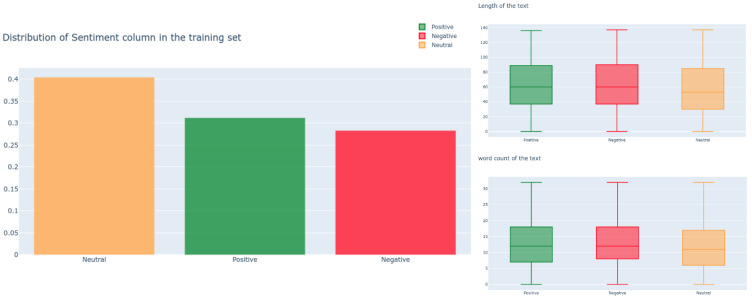
The left illustrates sentiment distributions, and the right is length of text and word count for each sentiment.

It is observed that there is a trivial class unbalancing between positive and negative samples (see Figure 7). Within a deep-learning context, we think this margin is negligible and that these can be considered as well-balanced samples. Additionally, the length of sentence and words are not useful features in distinguishing sentiment.

**Figure 8:**
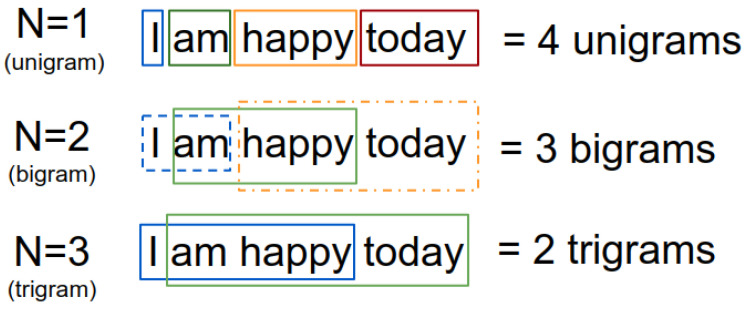
Examples of *N*-grams. Each box groups words depending on *N*.

Other statistics that we can extract from the corpus are N-grams, which are a contiguous sequence of N words/tokens/items from a given sample of text, as illustrated in Figure 8.

An input sentence, “I am happy today” produces four unigrams (“I”, “am”, “happy”, “today”) and three bigrams (“I am”, “am happy”, “happy today”). The unigrams themselves may be insufficient to capture context, but bigrams or trigrams contain more meaningful and contextually rich information. Based on this observation, we see what the most appearing N-grams in our dataset are.

**Figure 9:**
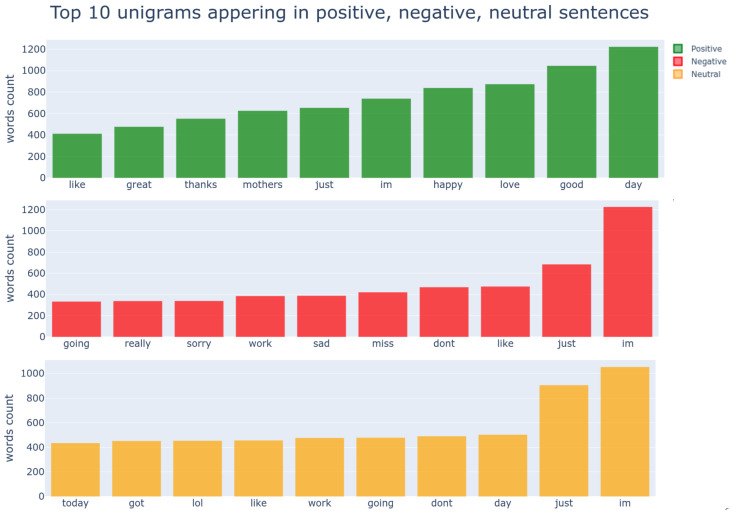
Statistic of top 10 unigrams in positive, negative, neutral sentiment. “im;’ and “just;’ appear in all sentiments with high counts, implying that leveraging unigrams meta information may cause confusion for models and lead to inferior performances.

**Figure 10:**
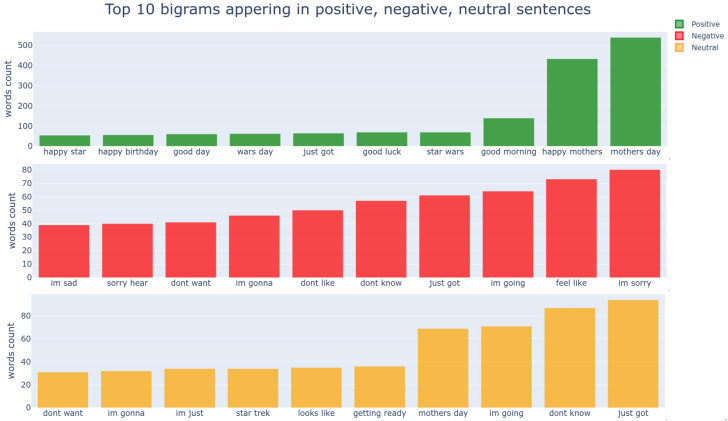
Top 10 bigrams appearing in positive, negative, neutral sentiment. By looking at top tier bigrams, they demonstrate better discrimination between sentiment than unigrams.

Figure 9 and Figure 10 show the frequency of uni- and bigrams in our training dataset. From the unigrams, one can find that positive words such as “good”, “great”, “like”, “happy”, and“love” ranked near the top and negative words; “sad”, “sorry”, and “dont” are top-ranked. These results are expected because the words in the same sentiment are closely located in the word embedding space. However, it is rather difficult to gauge solely based on unigrams (e.g., “im” appears in all sentiments).

The bigrams or trigrams can encapsulate a more distinguishable meaning, as shown in Figure 10. It is noticeable that “mothers day” is top-ranked, and we guess that this tweet dataset was sampled around the 9th of May.

From the EDA study, we conclude that the number of words or the length of sentences has a marginal impact in extracting sentiment, but the most important features are a contiguous sequence of words that align with our approach presented in Section 3.3.

### 4.4. Dataset Preparation

**Figure 11:**
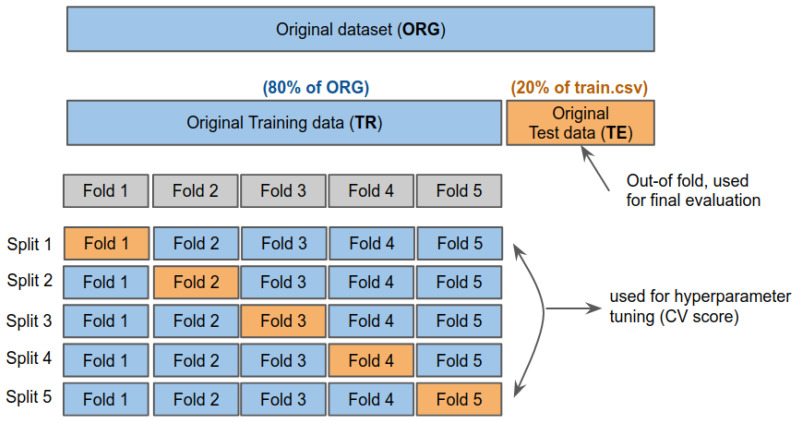
Five-fold cross-validation dataset split (reproduced of https://scikit-learn.org/stable/modules/cross_validation.html, accessed on 11 April 2021). We exploit this validation strategy across all experiments. Blue boxes are training set, and orange indicates validation/test set.

In this section, we present a dataset split strategy for model training and validation. As shown in Figure 5, we split the original dataset into 80% train and the rest, 20%, for testing. Although this split ratio is empirically chosen, it is commonly used in NLP and image-related deep learning tasks. Figure 11 illustrates train/test split and five folds CV split. We present further technical detail regarding CV in the following Section 5.2. The blue boxes are the training set, and the orange indicates the testing set. All 24 experiments presented in this paper follow this dataset split rule.

## 5. Model Training

The object of this section is training all 24 experiments (see Table 1). Given the the prepared dataset, this section presents the details of model training strategies such as Stratified K-fold cross-validation and training configuration.

### 5.1. Model Details

We trained three models—BERT, RoBERTa-base, and RoBERTa-large—for two different tasks: classification and coverage-based subsentence extraction. As mentioned earlier in Section 3.1, we used the original transformer architectures of BERT and RoBERTa and attached varying head layers that are placed on the top of each transformer, depending on the task. For the classification task, a fully connected layer (input dim: 768, output dim: 3) with a dropout (0.1) added, followed by the transformer model as shown in Table 6. The output of this network indicates how likely the input sentence is classified as one of the predefined sentiment classes (e.g., positive, negative, and neutral). This prediction is useful and boosts the subsentence extraction performance significantly (more detail regarding this is presented in the following section). This is mainly because the classification network is able to guide the subsentence extraction network toward the direction where it extracts subsentences from more correlated distributions. For example, suppose an input sentence is classified as positive. In that case, the subsentence extraction network is likely looking for positive words (e.g., good or fantastic) rather than negative words (bad or poor). In turn, this also leads to narrowing down the overall search space, which improves convergence time.

A coverage-based subsentence extraction network has slightly more complex architecture, as shown in Figure 4. The inputs of this are input sentence, classified sentiment, and coverage, which indicates the percentage of subsentence from the entire input sentence. The outputs are the indices of the starting and end words within the input sentence. We added three 1D Convolutional layers, followed by two fully-connected layers. Model hyperparameters are empirically selected, and details can be found in Table 6.

It is important to note that this coverage-based model makes use of the outputs of other networks such as sentiment and indices predictions. This is explained in more detail in the end-to-end entire pipeline section (Section 6.5).

### 5.2. K-Fold Cross Validation

It is common to use *K*-fold cross-validation (CV) for model validation, where K implies a model is trained on K−1 folds and tested with one remaining fold. Although this process is expensive (because it enforces *K* times model training phases), it is considered one of the most data-efficient and acceptable validation methods by machine learning communities. We thus apply five stratified CV (https://scikit-learn.org/stable/modules/generated/sklearn.model_selection.StratifiedKFold.html, accessed on 11 April 2021) to all 24 models to ensure properly equalised class distributions between training and test set of each fold.

### 5.3. Training Configuration

All model training was conducted on an RTX3090 GPU that has 24GB. BERT and RoBERTa-base took approximately one hour, and RoBERTa-large finished five-fold CV in two hours. In total, it took about 31 hours for the full model training. Training multiple models with such variants opens a tricky task because there are many things to consider, such as model parameters, logging, and validation. We developed a unified and automated NLP framework, BuilT (https://github.com/UoA-CARES/BuilT, accessed on 11 April 2021), which takes care of all necessary house-keeping and experiments above. Using BuilT, one can reproduce the same results we presented in this paper and develop other similar projects.

Adam optimiser (lr = 0.00003) is used with categorical Cross-Entropy Loss (CEL) [39] of smoothed label:

(1) $$y^{LS}=y\left(1-\alpha \right)+\frac{\alpha }{C}$$

where α is smoothness (e.g., when α is 0, then it is identical to CEL) and *C* is the number of label classes (our case *C* = 3). With the label smoothing, our loss is defined as:

(2) $$\begin{array}{cc} CE(x_{i},y_{i}^{LS})= & -\sum_{i=1}^{C}y_{i}^{LS}\log h_{\theta }\left(x_{i}\right) \end{array}$$

where $h_{\theta }\left(x_{i}\right)$ indicates the softmax output of our model parameterised by θ given *i*th input $x_{i}$ and is defined as

(3) $$\begin{array}{cc} h_{\theta }\left(x_{i}\right)= & \frac{\exp(x_{i})}{\sum_{j=1}^{C}\exp\left(x_{i}\right)} \end{array}$$

Finally, CEL can be compuated as

(4) $$\begin{array}{cc} CEL(x_{i},y_{i}^{LS})= & -\sum_{j=1}^{N}\left(\sum_{i=1}^{C}y_{i}^{LS}\log\frac{\exp(x_{i})}{\sum_{i=1}^{C}\exp\left(x_{i}\right)}\right) \end{array}$$

where *N* is the total number of training samples.

**Figure 12:**
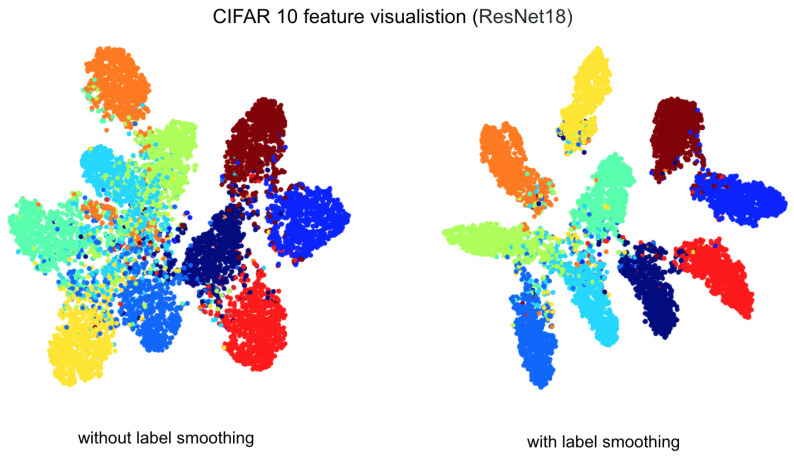
Feature visualisation using TSNE [40] without (**left**) and with label smoothing (**right**) for a classification task. Clearer inter-class boundaries can be observed with the label smoothing technique.

The use of this label smoothing [39] as exemplified in Figure 12 improved performance significantly in our case. This is mainly due to the fact that there is possible noise in the manual label (i.e., training set), such that this may force the model to learn inaccurate label strictly. Label smoothing eases the label as a function of α, making it less critical to this incorrect label. Note that this method does not always lead to superior results, and it depends on the quality of the dataset. Multistep learning rate scheduler (γ=0.1, stepping at 3, 4, 5) was used across all experiments.

## 6. Experimental Results

In this section, evaluation metrics used for evaluating the model performances (Jaccard score, AUC, and $F_{1}$ scores) are defined, followed by classification and coverage-based subsentence extraction models. Finally, the entire pipeline model evaluation is conducted for real-world applications and usage.

### 6.1. Evaluation Metrics

We used three metrics for different tasks because an individual task requires a task-specific metric. For the classification task, AUC and $F_{1}$ scores were used. These metrics can capture both precision and recall performance and are commonly accepted for the task. For the subsentence extraction task, Jaccard score is utilised for measuring the similarity between subsentences.

#### 6.1.1. Area-Under-the-Curve

**Figure 13:**
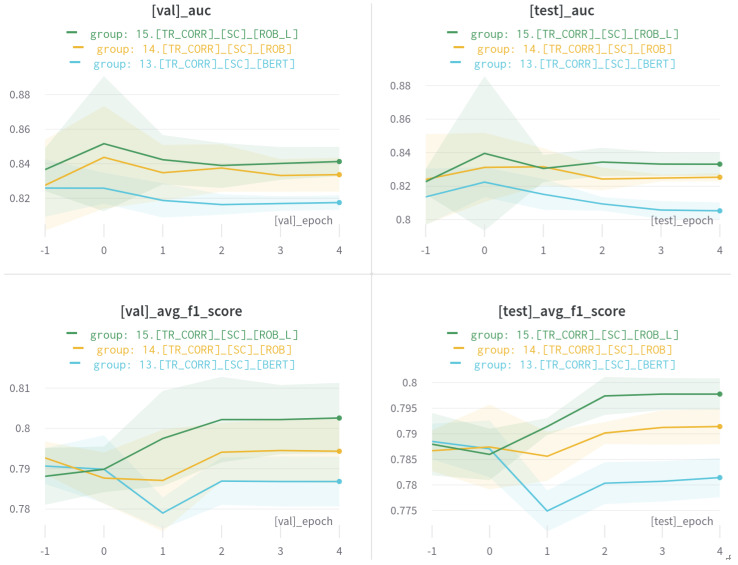
Index extraction results (AUC and F1 score) of three different models (i.e., Bert, RoBERTa-base, and RoBERTa-large). Shaded area is the standard deviation of 5 models from cross-validation, and solid lines denote average metrics displayed top of each plot.

AUC is the metric we use for gauging the performance of our classifier. As the name suggests, it measures the total area under the receiver operating characteristic curve (i.e., precision-recall curve). Precision (*P*) and recall (*R*) can be expressed as

(5) $$P=\frac{T_{P}}{T_{P}+F_{P}},\;\;\;R=\frac{T_{P}}{T_{P}+F_{N}}$$

where $T_{P}$ and $F_{P}$ are true/false positive, which stand for correct/incorrect classification and $F_{N}$ is false negative, that indicates mis-classification. From *P* and *R*, we can construct a performance curve by varying all possible thresholds as shown in Figure 13. Since this curve encapsulates all possible thresholds, it is independent of threshold tuning and a value closer to 1 indicates better performance. Due to these powerful and intuitive concepts, this AUC metric is one of the most widely accepted metrics in classification problems.

#### 6.1.2. Harmonic $F_{1}$ Score

Similarly, we also evaluate the proposed system using $F_{1}$ score, which can be expressed as

(6) $$F_{1}=\frac{T_{P}}{T_{P}+\frac{1}{2}(F_{P}+F_{N})}.$$

This harmonic $F_{1}$ score is one of the most widely accepted metrics in classification tasks because it reflects precision and recall performance.

#### 6.1.3. Jaccard Score for Subsentence Extraction

Jaccard score is a measure of similarity between two sentences. The higher the score, the more similar the two strings. In this paper, we tried to find the number of common tokens and divide it by the total number of unique tokens. This may be expressed as

(7) $$J_{score}\left(A,B\right)=\frac{\left|A\cap B\right|}{\left|A\cup B\right|}=\frac{\left|A\cap B\right|}{\left|A|+|B|-|A\cap B\right|}.$$

As an example, if we want to calculate a $J_{score}$ between sentence A, “Hello this is a really good wine” and B, “Hello, this is a really good wine.” (note that there is a trailing comma and period in sentence B), the score is 0.555. This metric is significantly lower than we anticipated based on the meanings of two input sentences (which are identical). Since we compare token level and split words based on space, “Hello” and “Hello,”, “wine” and “wine.” are treated as different words.

### 6.2. Model Ensemble

As mentioned in Section 5.2, we used five-fold CV for all 24 experiments, and this implies that we have five different models for each experiment. One of most popular methods to fuse these models is ensemble averaging, $\tilde{y}\left(x\right)$ and may be expressed as

(8) $$\tilde{y}\left(x\right)=\sum_{i=1}^{N}\alpha_{i}y_{i}\left(x\right).$$

*N* is the number of models to fuse (i.e., five in this case), and x is the input vector, $y_{i}$ is the prediction of the *i*th model, and $\alpha_{i}$ is the weight of the *i*th model. In our case, we used an equal average ensemble, which implies that $\alpha_{i}=\frac{1}{N}$. The equally-weighted ensemble strategy is rather simple yet powerful in dealing with model overfitting and in the presence of a noisy label (e.g., model voting). There is a wide range of ensemble options reported within the literature [41] on how to select the optimal weights for a particular task. A weakness in the use of an ensemble is that it requires O(N) time complexity for sequential processing or O(N) space for holding models in a machine’s memory.

Table 7 shows equally weighted model ensemble results. Most of experiments report performance boost. However, as depicted by Model 23 (bottom row), it is not always guaranteed to obtain superior performance with ensembled model. In such a case, other ensemble options can be applied.

### 6.3. Classification Results

Precise sentiment prediction is an essential front-end component by providing the scope of the subsentence search space. The goal of this section is to choose the most suitable sentiment classification network via intensive performance evaluation.

We performed six experiments with variations in datasets and models and decided to use the RoBERTa-base model. For visual inspection purposes, we only present three experiments in this section. The summary is shown in Table 8, and experimental results are displayed in Figure 13. The horizontal axis indicates epoch, and the vertical axis is the corresponding performance metric. The shaded area is the standard deviation of five models, with the mean value indicated by the solid line. We followed model naming conventions as defined Table 1 in Section 4. For instance, [TR_CORR]_[SC]_ROB] can be interpreted by the RoBERTa-base model trained on the corrected dataset for the sentiment classification task.

Overall, the RoBERTa-large model performed best for all validation and test experiments. However, this network consists of twice as many encoders as RoBERTa-base, which means theoretically about two times slower inferencing time. Our experiments prove that BERT and RoBERTa-base took about 4ms per sentence (100s for processing 5.4K test sentences) and 8ms for RoBERTa-large (220s). However, this is marginal to our real-time sentiment extraction task considering processing time and subsequent tasks such as SSML and multilabel extraction. Furthermore, the performance degradation with RoBERTa-base is 0.05∼0.1, which lies within our acceptable range. Therefore, we decided to use RoBERTa-base network for the following end-to-end pipeline (Section 6.5).

### 6.4. Coverage-Based Subsentence Extraction

**Figure 14:**
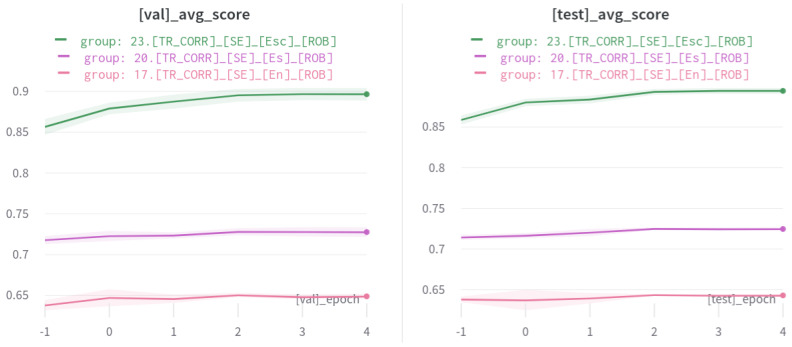
Jaccrad score performance evaluation of the proposed coverage model (green). Left and right plots display average scores of validation and test sets respectively. Shaded area is the standard deviation of 5 models. Model 17 (pink, with no additional meta data) performs least but Models 20 (magenta) and 23 (green) demonstrate impressive performance improvements with meta information.

Given the sentiment classification and subsentence extraction information, our coverage model aims to improve the performance by selectively refining span predictions. As shown in Figure 14, the coverage model outperforms other models with a large margin.

The 17.[TR_CORR]_[SE]_[En]_[ROB] model indicates performing subsentence extraction without sentiment information with RoBERTa-base backbone, and Model 20 is with that metadata. The presence of sentiment data boosts the overall Jaccard score by about 0.8. It is obvious that the extra encoding of data helped the model better localise correct indices (i.e., start and end indices of subsentence). Additional coverage information (Model 23) increased the performance to around 0.89, which impressively demonstrates our proposed approach. A summary is provided in Table 9. It is important to note that Model 23 assumes that sentiment information is also given, which is not always true in real-world scenarios, where only an input sentence is provided. We are then required to predict not only coverage, but sentiment as well in the pipeline. Therefore, we present the pipeline experiment results in the following section.

### 6.5. End-to-End Pipeline Evaluation Results

**Figure 15:**
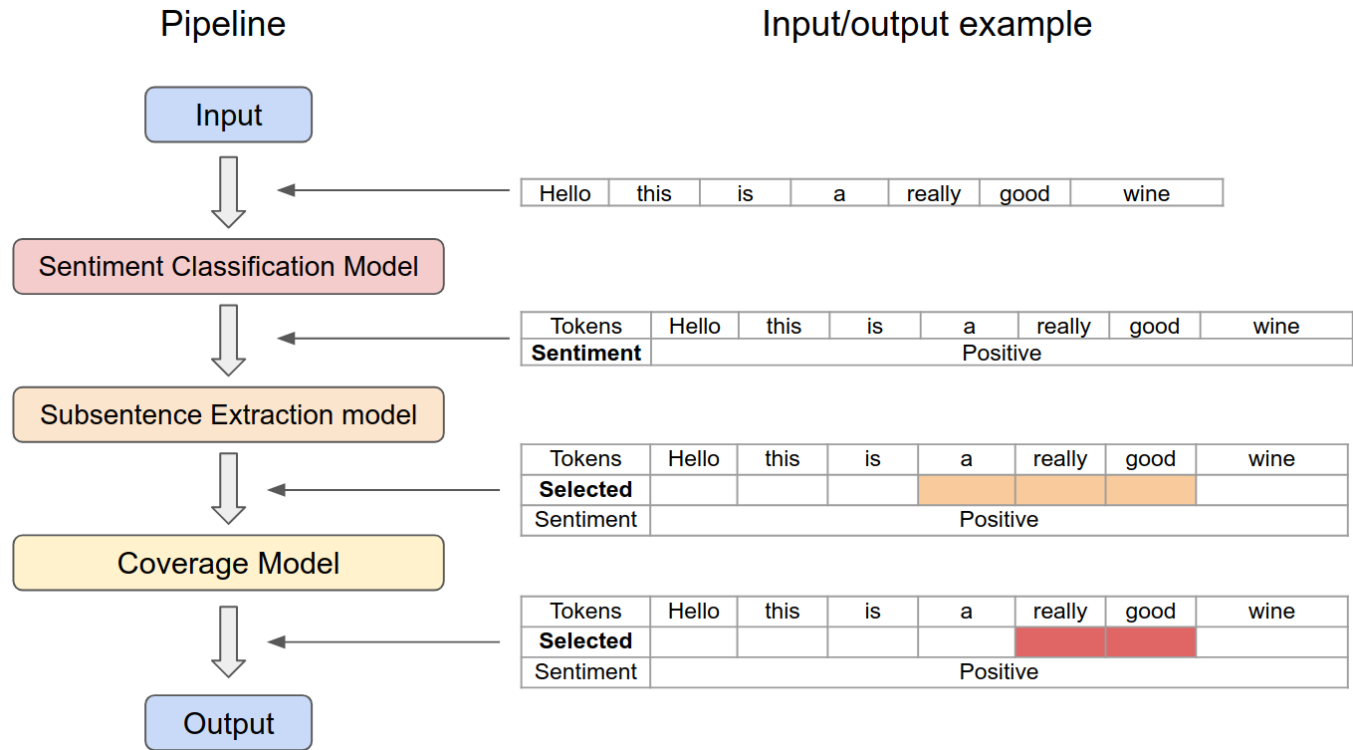
Sentiment and subsentence prediction pipeline. This diagram is manually illustrated with an example just to highlight refining procedure with the proposed coverage model.

In order to use the proposed approach in real-world scenarios such as HRI, one must perform sentiment and subsentence predictions sequentially. Based on previous experiments, we developed a pipeline predicting sentiment using 14.[TR_CORR]_[SC]_[ROB] and fed the sentiment to 20.[TR_CORR]_[SE]_[Es]_[ROB] for the initial subsentence extraction and finally, 23.[TR_CORR]_[SE]_[Esc]_[ROB] for refined subsentence prediction. Figure 15 illustrates the pipeline with an example input sentence. In this example, it is observed that our coverage model can refine the subsentence range so that it leads to superior results.

We tested the proposed pipeline with four cases conditioned by a sentiment and coverage model, as shown in Table 10. From the table, the left column indicates the source of sentiment meta information, and the top row is the presence of the coverage model. The model with coverage and without it for inferencing 5.4k sentences achieved Jaccard scores of **0.7265**, and 0.7256, respectively. The proposed coverage model slightly improves the overall performance of both cases. Note that the result shown in the middle row (i.e., Model 20) is inferior to the bottom, which assumes the sentiment label is given from the manual label. This is caused by error propagation in the sentiment model throughout the entire pipeline.

### 6.6. Class Activation Mapping

**Figure 16:**
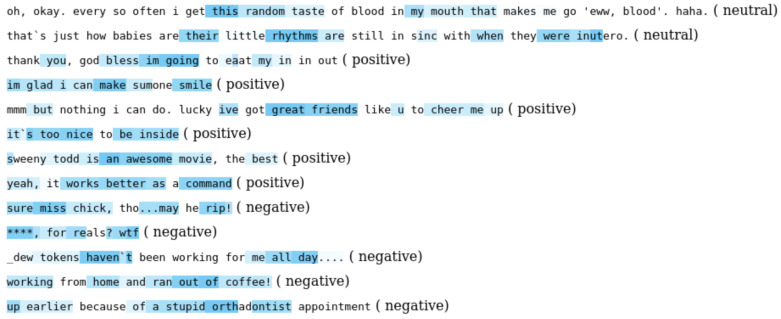
Examples of multilabel attention. Each row is an input sentence with highlighted predicted activation. The darker blue represents the higher probability. It is important to note that our framework is capable of predicting multiple labels from one sentence, which can result in better machine–human interaction experience.

In performing the sentiment classification task, networks often only output classes with the corresponding probability. However, they also learned about what caused that output in their internal layers. For example, in the image classification task, an activation map can be extracted from one of the internal layers that highlight a region of interest in an image. This technique is useful for debugging purposes and, more importantly, valuable feature extraction. Analogous to this, in an NLP context, we can use a Class Activation Mapping (CAM) technique to extract the activation of a word that indicates how much the word contributed to the classification task. Figure 16 illustrates activation of words identifying contribution to sentiment classification. For example, the words “smile”, “awesome” and “great” show stronger responses (the darker blue is the higher probability) in the positive sentence. Another useful aspect is that multi-activation can be leveraged as information-rich features or input for speech synthesis markup language (SSML) for more vivid and enhanced natural human–machine interaction.

## 7. Discussion and Limitations

This section reports the issues and limitations that we encountered while developing the sentiment and subsentence extraction framework and when conducting the experiments.

We encountered a common machine learning problem; high variance or overfitting. Even though the dataset is well-balanced between positive and negative sentiment, neutral takes a large portion of the dataset that we bypassed for subsentence extraction (i.e., an input sentence with neutral sentiment is identical to its subsentence). This operation was intentionally performed due to the dataset reflecting this pattern. In turn, this affects the coverage model to avoid shrinking its coverage range. This is why our coverage model has a lack of ability to shrink, rather than expand. Another reason may be that the average length of subsentences in the dataset is relatively long with respect to input sentence (e.g., ≥0.5) so that the model can only learn to expand a coverage span. This depends on how the dataset is labelled by human labellers. To address this, we are investigating adaptive coverage span methods by making use of different means such as a weighted cross-entropy loss function that treats each class with varying weights that are estimated from class distributions, as well as introducing outlier rejection that filters out samples that have large margins with respect to their class distributions.

## 8. Conclusions

In this paper, we propose a real-time and highly accurate sentence-to-sentiment framework. Based upon the recent success in NLP using transformer models, we designed a coverage-based subsentence extraction model that outperforms other work with a large margin. This model learns how to further refine a span of subsentence in a recursive manner and, in turn, increases performance. We intensively evaluated the performance of 24 models by utilising acceptable metrics and methods such as AUC, and $F_{1}$ scores for classification and Jaccard for subsentence extraction and stratified five-fold CV. The use of meta-information significantly helps models predict accurate subsentences (e.g., 0.643 to 0.724 Jaccard score), and coverage encoding impressively improves performance by a large margin (0.8944). We also demonstrate that an ensemble of five models boosts the performance and present experiments with the entire pipeline framework that takes only a sentence as input to be used for many real-world useful applications.

## Figures and Tables

**Table 1:** All experiments’ names and descriptions. Experiment naming convention used across all sections is [Dataset]_[Task]_ [EXT]_[Transformer].

Experiment Name	Dataset	Task	Encoding	Transformer
1.[TR]_[SC]_[BERT]	Original train data	Classification	N/A	BERT
2.[TR]_[SC]_[ROB]	Original train data	Classification	N/A	RoBERTa-base
3.[TR]_[SC]_[ROB_L]	Original train data	Classification	N/A	RoBERTa-large
4.[TR_CORR]_[SC]_[BERT]	Corrected train data	Classification	N/A	BERT
5.[TR_CORR]_[SC]_[ROB]	Corrected train data	Classification	N/A	RoBERTa-base
6.[TR_CORR]_[SC]_[ROB_L]	Corrected train data	Classification	N/A	RoBERTa-large
7.[TR]_[SE]_[En]_[BERT]	Original train data	Subsentence Extraction	None	BERT
8.[TR]_[SE]_[Es]_[BERT]	Original train data	Subsentence Extraction	Sentiment	BERT
9.[TR]_[SE]_[Esc]_[BERT]	Original train data	Subsentence Extraction	Sentiment + coverage	BERT
10.[TR]_[SE]_[En]_[ROB]	Original train data	Subsentence Extraction	None	RoBERTa-base
11.[TR]_[SE]_[Es]_[ROB]	Original train data	Subsentence Extraction	Sentiment	RoBERTa-base
12.[TR]_[SE]_[Esc]_[ROB]	Original train data	Subsentence Extraction	Sentiment + coverage	RoBERTa-base
13.[TR]_[SE]_[En]_[ROB_L]	Original train data	Subsentence Extraction	None	RoBERTa-large
14.[TR]_[SE]_[Es]_[ROB_L]	Original train data	Subsentence Extraction	Sentiment	RoBERTa-large
15.[TR]_[SE]_[Esc]_[ROB_L]	Original train data	Subsentence Extraction	Sentiment + coverage	RoBERTa-large
16.[TR_CORR]_[SE]_[En]_[BERT]	Corrected train data	Subsentence Extraction	None	BERT
17.[TR_CORR]_[SE]_[Es]_[BERT]	Corrected train data	Subsentence Extraction	Sentiment	BERT
18.[TR_CORR]_[SE]_[Esc]_[BERT]	Corrected train data	Subsentence Extraction	Sentiment + coverage	BERT
19.[TR_CORR]_[SE]_[En]_[ROB]	Corrected train data	Subsentence Extraction	None	RoBERTa-base
20.[TR_CORR]_[SE]_[Es]_[ROB]	Corrected train data	Subsentence Extraction	Sentiment	RoBERTa-base
21.[TR_CORR]_[SE]_[Esc]_[ROB]	Corrected train data	Subsentence Extraction	Sentiment + coverage	RoBERTa-base
22.[TR_CORR]_[SE]_[En]_[ROB_L]	Corrected train data	Subsentence Extraction	None	RoBERTa-large
23.[TR_CORR]_[SE]_[Es]_[ROB_L]	Corrected train data	Subsentence Extraction	Sentiment	RoBERTa-large
24.[TR_CORR]_[SE]_[Esc]_[ROB_L]	Corrected train data	Subsentence Extraction	Sentiment + coverage	RoBERTa-large

**Table 2:** An exemplified input data and its tokenisation.

Input Sentence = “Hello This Is a Really Good Wine”						Subsentence = “Really Good”				Sentiment = Positive				
tokens =	<s>	Hello	this	is	a	really	good	wine	</s>	</s>	positive	</s>	<pad>	<pad>
input_ids =	0	812	991	2192	12	3854	202	19292	2	2	1029	2	1	1
attention_mask =	1	1	1	1	1	1	1	1	1	1	1	1	0	0
start_token =	0	0	0	0	0	1	0	0	0	0	0	0	0	0
end_token =	0	0	0	0	0	0	1	0	0	0	0	0	0	0

**Table 3:** Tweet sentiment dataset.

	Total	Unique	Positive	Negative	Neutral
**Tweet**	27,480	27,480	8582 (31.23%)	7781 (28.32%)	11,117 (40.45%)
**Selected text**	27,480	22,463	N/A	N/A	N/A

**Table 4:** Five samples from training dataset with meta information.

	textId	Text	Selected_TEXT	Sentiment
**0**	cb774db0d1	I’d have responded, if I were going	I’d have responded, if I were going	neutral
**1**	549e992a42	Sooo SAD I will miss you here in San Diego!!!	Sooo SAD	negative
**2**	088c60f138	my boss is bullying me...	bullying me	negative
**3**	9642c003ef	what interview! leave me alone	leave me alone	negative
**4**	358bd9e861	Sons of ****, why could not they put them on t...	Sons of ****,	negative

**Table 5:** Examples of incorrect and corrected selected text (right most column). selected_text and sentiment columns are manually labelled meta information which are corrupted due to special characters.

Text	Selected_TEXT	Sentiment	Corrected_Selected_Text
is back home now gonna miss every one	onna	negative	miss
He’s awesome... Have you worked with them before...	s awesome	positive	awesome.
hey mia! totally adore your music. when...	y adore	positive	adore
Nice to see you tweeting! It’s Sunday 10th...	e nice	positive	nice
#lichfield #tweetup sounds like fun Hope to...	p sounds like fun	positive	sounds like fun
nite nite bday girl have fun at concert	e fun	positive	fun
HaHa I know, I cant handle the fame! and thank you!	d thank you!	positive	thank you!

**Table 6:** Emotion classification and subsentence extraction network architecture.

Task	Layer	Input Output	Task	Layer	Input Output
	RoBERTa	Input sentence 768		RoBERTa	Input + Attention Mask + Sentiment + Coverage 768
	Dropout	0.1		Dropout	0.3
	FC ^a^	768		Conv1D	768
Classification		3	Covereage-based subsentence extraction		256
				Conv1D	256
					128
				Conv1D	128
					64
				FC	64
					32
				FC	32
					2

^a^ Fully-connected.

**Table 7:** Equally weighted ensemble result summary.

	**Classification**	
**Trained Model from Experiment**	**Average F1**	**Ensemble F1**
13.[TR_CORR]_[SC]_[BERT]↑ ^a^	0.7890	0.7957
14.[TR_CORR]_[SC]_[ROB]↑	0.7921	0.8026
15.[TR_CORR]_[SC]_[ROB_L]↑	0.7973	**0.8084**
	**Subsentence Extraction**	
**Trained Model from Experiment**	**Average Jaccard**	**Ensemble Jaccard**
17.[TR_CORR]_[SE]_[En]_[ROB]↑	0.6457	0.6474
20.[TR_CORR]_[SE]_[Es]_[ROB]↑	0.7249	0.7257
23.[TR_CORR]_[SE]_[Esc]_[ROB]↑	**0.8944**	0.8900

^a^ the values closer to 1 indicate better performance.

**Table 8:** Sentiment classification results summary.

	13.[TR_CORR]_[SC]_[BERT]		14.[TR_CORR]_[SC]_[ROB] ^a^		15.[TR_CORR]_[SC]_[ROB_L]	
	Val	Test	Val	Test	Val	Test
Average AUC↑	0.8175	0.8053	0.8336	0.8253	**0.8412**	0.8331
Average $F_{1}$↑	0.7868	0.7978	0.7943	0.7914	**0.8026**	0.7814

^a^ This is the model used for the subsequent coverage model.

**Table 9:** Coverage-based subsentence extraction results summary.

Model	Val. Jaccard	Test Jaccard
17.[TR_CORR]_[SE]_[En]_[ROB]	0.649	0.643
20.[TR_CORR]_[SE]_[Es]_[ROB]	0.7277	0.7247
23.[TR_CORR]_[SE]_[Esc]_[ROB]	**0.8965**	**0.8944**

**Table 10:** Pipeline subsentence extraction results summary.

Source of Sentiment	With Coverage	W/O Coverage
Classifier prediction	**0.6401**	0.6392
Manual label	**0.7265**	0.7256

## Data Availability

https://github.com/UoA-CARES/BuilT, accessed on 11 April 2021.

## Abbreviations

The following abbreviations are used in this manuscript

AI	Artificial Intelligence
AUC	Area-Under-the Curve
BERT	Bidirectional Encoder Representations from Transformers
BPE	Byte-Pair Encoding
CAM	Class Activation Mapping
CEL	Cross-Entropy Loss
CV	Cross-Validation
EDA	Exploratory Data Analysis52
GLUE	General Language Understanding Evaluation
GPT-3	Generative Pre-trained63Transformer 3
HRI	Human–Robot Interaction
NLP	Natural Language Processing
NSP	Next Sentence Prediction
RoBERTa	A Robustly Optimized BERT Pretraining Approach
S2E	Sentence To Emotion
SOTA	State-Of-The-Art
SSML	Speech Synthesis Markup Language
SQuAD	Stanford Question Answering Dataset
